# The Role of Cystine/Glutamate Antiporter SLC7A11/xCT in the Pathophysiology of Cancer

**DOI:** 10.3389/fonc.2022.858462

**Published:** 2022-02-23

**Authors:** Nidhi Jyotsana, Kenny T. Ta, Kathleen E. DelGiorno

**Affiliations:** ^1^ Department of Cell and Developmental Biology, Vanderbilt University, Nashville, TN, United States; ^2^ Vanderbilt-Ingram Cancer Center, Vanderbilt University Medical Center, Nashville, TN, United States; ^3^ Vanderbilt Digestive Disease Research Center, Vanderbilt University Medical Center, Nashville, TN, United States; ^4^ Epithelial Biology Center, Vanderbilt University Medical Center, Nashville, TN, United States

**Keywords:** SLC7A11 (xCT), metabolism, cysteine (Cys), gastrointestinal tract, ferroptosis, oxidative stress, Cancer therapy

## Abstract

SLC7A11/xCT is an antiporter that mediates the uptake of extracellular cystine in exchange for glutamate. Cystine is reduced to cysteine, which is a rate-limiting precursor in glutathione synthesis; a process that protects cells from oxidative stress and is, therefore, critical to cell growth, proliferation, and metabolism. SLC7A11 is expressed in different tissues and plays diverse functional roles in the pathophysiology of various diseases, including cancer, by regulating the processes of redox homeostasis, metabolic flexibility/nutrient dependency, immune system function, and ferroptosis. SLC7A11 expression is associated with poor prognosis and drug resistance in cancer and, therefore, represents an important therapeutic target. In this review, we discuss the molecular functions of SLC7A11 in normal *versus* diseased tissues, with a special focus on how it regulates gastrointestinal cancers. Further, we summarize current therapeutic strategies targeting SLC7A11 as well as novel avenues for treatment.

## Introduction

Amino acid metabolism is altered in many cancers, reflecting cancer cell uptake and altered metabolic needs, and targeting these pathways is becoming an attractive approach to patient therapy (1). The amino acid cysteine plays important roles in protein synthesis and in maintaining redox balance (2–4). Intracellular cysteine can be produced *de novo* or recycled through protein degradation (5, 6). However, under conditions of oxidative stress, such as in cancer, *de novo* biosynthesis or a catabolic supply of cysteine is not sufficient to meet the high demand for antioxidant synthesis. Therefore, most cancer cells rely on nutrient transporters that can import extracellular cystine (the oxidized dimer form of cysteine) (7, 8). Solute Carrier Family 7 Member 11 (SLC7A11) or xCT is the functional light chain subunit of system ${\text{x}}_{\text{c}}^{-}$ , which is a sodium-independent, chloride-dependent, anionic L-cystine/L-glutamate antiporter on the cell surface (9, 10). The SLC7A11 protein requires either of the two heavy chain subunits of SLC3A2 to import extracellular cystine in exchange for intracellular glutamate, at a molar ratio of 1:1 (11, 12). The *SLC7A11* gene in humans is located on chromosome 4 and the SLC7A11 protein has orthologs in all vertebrates (4, 11). With 12 transmembrane domains, the N- and C- termini of the SLC7A11 protein reside in the cytoplasm (4). Unlike SLC3A2, which is a chaperone protein for many other light subunits of heterotrimeric amino acid transporter systems, SLC7A11 is specific for system ${\text{x}}_{\text{c}}^{-}$ and, therefore, serves as the primary transporter for cystine and glutamate (4, 11, 13).

**Figure 1:**
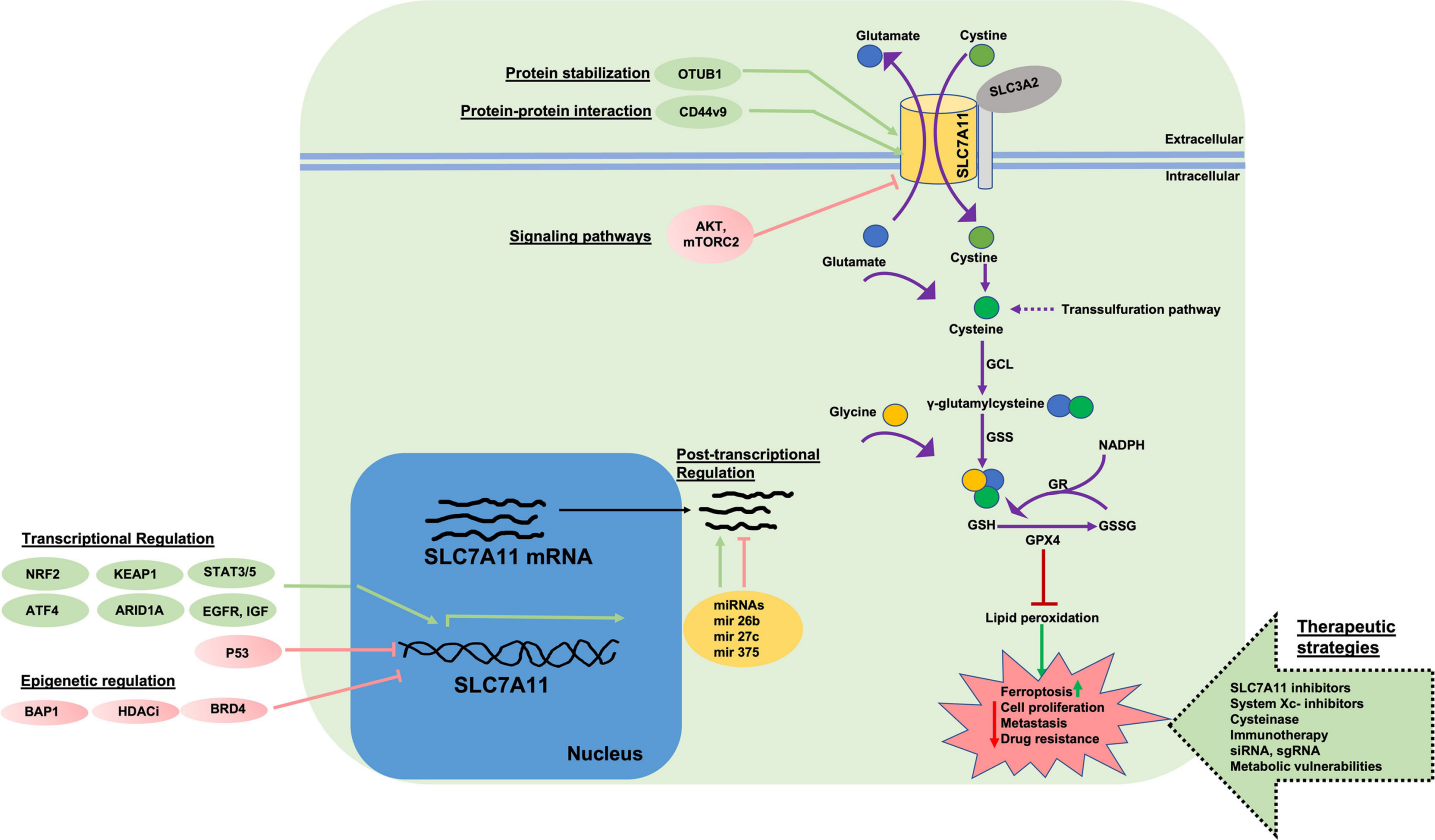
Function, Regulation, and Targeting of SLC7A11. System ${\text{x}}_{\text{c}}^{-}$ is a 1:1 cystine/glutamate exchange antiporter system comprised of light chain subunit SLC7A11 and heavy chain subunit (oligosaccharide-bound, gray circle) SLC3A2. SLC7A11 imports extracellular cystine which is reduced to cysteine through a NADPH-consuming reaction. Cysteine is utilized in conjugation with glutamate, by γ-GCL, to form γ-glutamylcysteine. In the next step, GSH tripeptide is produced *via* GS enzyme in conjugation with glycine. Intracellular cysteine can be synthesized by transsulfuration. Reduced GSH serves as a cofactor for enzymes such a GPX4 that protect cells from lipid peroxidation or ROS-induced damage. GSH is oxidized to GSSG *via* GPX4 and is recycled back to GSH at the expense of NADPH by GR enzyme. Accumulation of lipid peroxides leads to ferroptosis. In cells with high SLC7A11 levels and cystine import, GPX4 reduces lipid peroxidation and ferroptosis at the expense of GSH and protects cells from damage, allowing for cell proliferation, survival, and drug resistance. SLC7A11 is regulated at multiple levels. Transcriptional activation of SLC7A11 is induced by factors such as NRF2, ATF4, KEAP1, ARID1, STAT3/5 and is negatively regulated by p53 and ATF3. Epigenetic regulators (BAP1, BRD4 and HDAC inhibitors) negatively regulate SLC7A11 expression at the chromatin level. Post-transcriptionally, SLC7A11 is regulated by various miRNAs. Post-translationally, SLC7A11 protein is stabilized *via* protein-protein interactions (OTUB1, CD44v, CD44v9) which complex with SLC7A11 and inhibit proteasomal degradation. mTORC2 and AKT signaling inhibit SLC7A11 activity *via* phosphorylation. EGFR and IGF interact with SLC7A11 and maintain localization at the plasma membrane. SLC7A11 protein and function can be targeted *via* 1) direct inhibitors (e.g. erastin), 2) cysteinase, 3) immunotherapy, 4) targeting SLC7A11 *via* sgRNA and siRNA, 5) targeting metabolic vulnerabilities in cells with high SLC7A11 levels. ROS, Reactive oxygen species; GCL, γ-glutamylcysteine synthetase; GSS, glutathione synthetase; GPX4, glutathione peroxidase 4; GR, glutathione reductase; GSH, reduced glutathione; GSSG, oxidized glutathione; IKE, imidazole ketone erastin; GLUT, glucose transporter; sgRNA, single guide RNA; siRNA, small interfering RNA; ATF4, activating transcription factor 4; KEAP1, Kelch-like ECH associated protein-1; NRF2, nuclear factor erythroid 2-related factor 2; p53, tumor protein p53; ATF3, activating transcription factor 3; BAP1, BRCA1 associated protein-1; OTUB1, OTU deubiquitinase; ubiquitin aldehyde binding 1; mTORC2, mechanistic target of rapamaycin complex 2; miRNA, micro-RNA; HDACi, histone deacetylase inhibitors; EGFR, EGF receptor; IGF, insulin growth factor; ARID1A, AT-rich interactive domain-containing protein 1A; BRD4, bromodomain containing protein 4; NADPH, nicotinamide adenine dinucleotide phosphate.

Extracellular cystine is imported into the cell through SLC7A11 and is converted to cysteine through a NADPH-consuming reduction reaction in the highly reducing atmosphere of the cytosol. Cysteine is further utilized to synthesize glutathione (GSH), a tripeptide, through a two-step process (1. Formation of γ-glutamylcysteine from cysteine and glutamate catalyzed by γ-glutamylcysteine synthatase; 2. Formation of GSH from γ-glutamylcysteine catalyzed by glutathione synthetase) (**Figure 1**) (4, 14, 15). SLC7A11 is a major regulator of metabolic reprogramming in normal and cancer cells. This mainly includes effects on nutrient dependency (glucose metabolism and glutamine dependency), and intracellular redox balance. Extracellular cysteine is also imported into cells through an alanine-serine-cysteine transporter and can be synthesized *de novo via* a trans-sulfuration pathway in some tissues (for example, liver, kidney, and pancreas), however, SLC7A11 remains an important transporter for cancer cells that are largely dependent on extracellular cystine for survival (6, 16). In normal tissues, SLC7A11 is primarily expressed in the brain with low levels in most other tissues (17, 18). *Slc7a11* knockout mice are viable, fertile, healthy in appearance with no evident deleterious phenotype, and live a normal lifespan (19, 20). SLC7A11 is found upregulated in various human cancers (2, 4, 8, 21–23). It is likely that *de novo* synthesis of cysteine and/or its import *via* transporters other than SLC7A11 can fulfill the requirement of intracellular cysteine in normal cells or tissues in the absence of SLC7A11. Therefore, the dispensability of SLC7A11 in normal systems and its high expression in various cancers makes it an attractive therapeutic target for cancer treatment.

## Regulation of SLC7A11 at the Molecular Level

The expression of SLC7A11 can be modulated by various stress-inducing conditions (metabolic stress, amino acid starvation, genotoxic stress, hypoxia, viral infection) through transcription factors, epigenetic regulators, protein stability, interaction with other proteins, post transcriptional regulation, post-translational regulation, and transporter activity (**Figure 1**) (4). Studies have identified two transcription factors, nuclear factor erythroid 2-realted factor 2 (NRF2) and activating transcription factor 4 (ATF4), that regulate stress-induced SLC7A11 transcription (24–27). Under conditions of cell stress, the proteasomal degradation of NRF2 is impaired resulting in its stabilization and translocation to the nucleus. In the nucleus, NRF2 binds to antioxidant response elements (AREs) present in gene promoter regions and regulates the transcription of several antioxidant response-associated target genes including SLC7A11. The translation of ATF4 mRNA is enhanced under cell stress conditions but is otherwise repressed due to the presence of untranslated open reading frames (uORFs) located in the 5’ untranslated region of ATF4 mRNA. Stress, including amino acid deprivation, causes phosphorylated eukaryotic initiation factor 2α (eIF2α) to inhibit the translation of many mRNAs including ATF4 uORFs, liberating ATF4 translation and increasing ATF4 protein production. ATF4 further binds to amino acid response elements (AAREs) in promoter regions of genes like *SLC7A11* which allow the cells to adapt and respond to stress (28). For example, we and others have observed that under cystine starvation conditions, pancreatic cancer cells upregulate SLC7A11 expression as a stress response (25). Under these conditions, NRF2 and ATF4 interact and cooperatively regulate SLC7A11 expression. In contrast to NRF2 and ATF4, p53 has been shown to repress SLC7A11 expression, however, the mechanism is unclear (29, 30). Recently, NRF2 and ATF4 deficiency was shown to reduce SLC7A11 levels resulting in improved cancer cell survival under low glucose conditions. Restoration of SLC7A11 in NRF2- or ATF4- deficient cells re-sensitizes them to glucose starvation. Similarly, NRF2 activation in cancer cells results in increased sensitivity towards glutamine starvation and glutaminase inhibition, potentially due to increased SLC7A11-mediated glutamate export (31).

In addition to the transcriptional regulation of SLC7A11 under conditions of cell stress, SLC7A11 is also regulated *via* post-transcriptional mechanisms (5, 32). For example, SLC7A11 mRNA can be degraded by nonsense-mediated mRNA decay or negatively regulated by microRNAs (miR-27a, miR-26b, miR-375) (33–35). Histone modifications and chromatin remodeling also control SLC7A11 expression (5). BAP1 (BRCA1 associated protein-1) deubiquitates histone 2A mono-ubiquitination (H2Aub) at lysine-119 on the *SLC7A11* gene and is associated with the transcriptional repression of SLC7A11 and ferroptosis induction. Additionally, BAP1 deficiency in cancer cells is associated with SLC7A11 upregulation and ferroptosis resistance (36–38). Polycomb repressive complex 1 (PRC1) is a ubiquitin ligase that mediates histone 2A mono-ubiquitination at the SLC7A11 promoter and represses expression (31, 38). The SWI/SNF complex is involved in chromatin remodeling. ARID1A, a component of the SWI/SNF complex binds to the SLC7A11 promoter and promotes NRF2 mediated transcriptional activation. ARID1A deficiency is associated with impaired cystine uptake and ROS induction (39, 40).

Finally, interaction with other proteins and post-translational modifications can affect the expression and activity of SLC7A11. For example, SLC3A2 is required to maintain SLC7A11 stability (13). CD44 variant (CD44v) isoforms interact with and stabilize SLC7A11 in cancer cells. CD44v maintains SLC7A11 protein stability and CD44v deficiency compromises the cell surface localization of SLC7A11, resulting in ROS induction and inhibition of tumor formation (41, 42). It was found through collaborative stable isotope labeling in cell culture (SILAC) and mass spectrometric analysis of glioblastoma cells modified to overexpress SLC7A11, that mTORC2 (mammalian target of rapamycin complex 2) binds to the N-terminal cytoplasmic tail of SLC7A11 and inhibits its transport activity *via* phosphorylation at serine 26 (43). Epidermal growth factor receptor (EGFR) was shown to interact with SLC7A11 protein to maintain its localization at the cell membrane; increased cystine uptake and enhanced glutamate export associated with tumor growth and invasiveness has been observed in EGFR-expressing glioma cells (44).

## The Functional Role of SLC7A11 in Cellular Stress and Carcinogenesis

### Nutrient Dependency and Metabolic Flexibility

In comparison to normal cells, cancer cells differentially depend on certain nutrients for their survival and growth. Under certain nutrient deficient conditions, cancer cells undergo cell death whereas normal healthy cells survive because they are more metabolically flexible (45, 46). Understanding the mechanisms behind the limited metabolic flexibility, or nutrient dependency, of cancer cells may allow for targeted strategies that effectively kill cancer cells while sparing healthy cells. Glucose and glutamine are the main nutrients that provide energy for the biosynthetic machinery and various metabolic processes in most cells. Under glucose-limited conditions, for example, cells often increase their dependence on glutamine metabolism for survival (45, 47–49).

**Figure 2:**
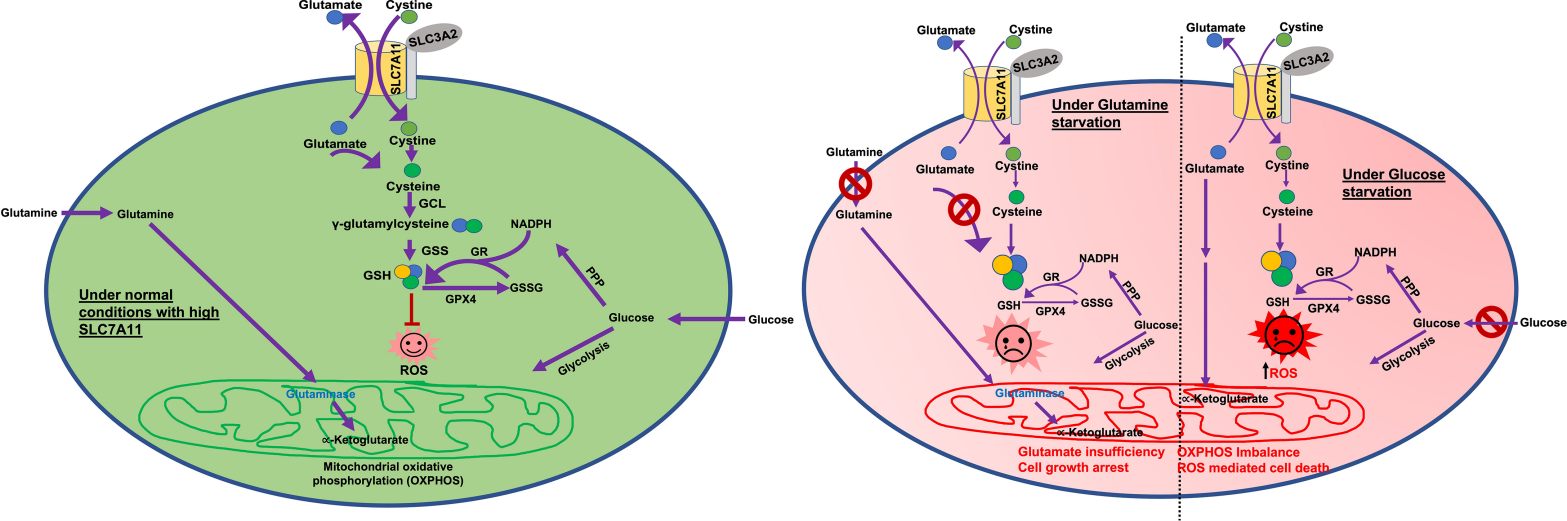
Glucose and glutamine dependency in SLC7A11^high^ cancers. Glutamine is the major precursor for glutamate. Glucose is the main supplier of 1) carbon for the TCA cycle and 2) NADPH for GSH production. Under normal conditions (green circle) SLC7A11^high^ cells exchange higher amounts of glutamate with cystine. Large amounts of imported cystine are converted to cysteine for GSH production and ROS detoxification. However, higher export of glutamate leaves cells more dependent on glucose and/or glutamine to feed the TCA cycle and oxidative phosphorylation/mitochondrial respiration. Under glucose deprived conditions (red circle-right half), SLC7A11^high^ cells lack the required levels of carbon. Higher cystine import *via* SLC7A11 leads to higher GSH synthesis and a glucose-deficiency induced depletion in NADPH. This results in ROS induction and enhanced cell death. Under glutamine deprived conditions (red circle-left half) in SLC7A11^high^ cells, higher glutamate export results in depletion of intracellular glutamate which feeds into the TCA cycle. This drives enhanced import of glutamine and glutaminase activity to synthesize glutamate, resulting in glutamine dependency. Therefore, under glutamine starvation conditions, intracellular glutamate is inadequate to fuel the TCA cycle, leading to growth arrest. ROS, Reactive oxygen species; GSH, reduced glutathione; GSS, glutathione synthetase; GPX4, glutathione peroxidase 4; GR, glutathione reductase; GSSG, oxidized glutathione; PPP, pentose phosphate pathway; TCA cycle, tricarboxylic acid cycle; αKG, α-ketoglutarate; NADPH, nicotinamide adenine dinucleotide phosphate.

SLC7A11 mediated cystine uptake is crucial to main redox homeostasis and biomass incorporation in cells (2, 4). Cancer cells increase SLC7A11 levels to enhance cystine import and maintain ROS homeostasis (8, 50, 51) (**Figure 2**). Unlike glucose and glutamine, which are taken up by their corresponding transporters, imported cystine must first be reduced to cysteine (52, 53). Because of the 1:1 exchange of cystine and glutamate across the cell membrane, a large amount of glutamate is exported with an upregulation of SLC7A11 (53). Glutamine is the most abundant amino acid in plasma. Once imported, glutamine is converted into glutamate *via* glutaminase and serves as a precursor for GSH synthesis, in addition to a role in the tricarboxylic acid (TCA) cycle (via conversion to α-ketoglutarate) (50, 51). Interestingly, high cystine levels in culture medium were observed to induce cystine uptake in cancer cells leading to greater glutamate export and, therefore, increased glutamine dependency (54). It has been shown that basal and claudin-low triple-negative breast cancer (TNBC) cell lines are more dependent on glutamine. These cells lines are SLC7A11-high and import more cystine than other breast cancer subtype cell lines. Treatment with sulfasalazine, a pharmacological inhibitor of SLC7A11, attenuates the growth of xenograft tumors derived from these cell lines. This suggests that SLC7A11-high cancers, like TNBCs, are more dependent on glutamine (55). This is likely because they need more glutamate to exchange for cystine due to higher SLC7A11 levels. Similarly, several pancreatic cancer cell lines (for example, PANC-1, BxPC-3 or HPAC) are glutamine dependent and results from Badgley et al. show that glutamine dependent pancreatic cancer cells are more sensitive to pharmacological and genetic inhibition of SLC7A11 (56).

Many studies indicate that several SLC7A11-high cancers are sensitive to glucose deprivation, however the exact mechanism here remains an active area of research. The loss of SLC7A11 and SLC3A2 has been shown to promote cancer cell survival under glucose starvation conditions and SLC7A11 overexpression promotes glucose starvation-induced cell death and vice versa (50, 51). Additionally, glucose deprivation was observed to induce SLC7A11 expression in cancer cells. As both glutamine and glucose are important for the TCA cycle, it has been speculated that enhanced SLC7A11 export of glutamate in cancer cells makes them more dependent on glucose to replenish the TCA cycle (5, 50, 51). However, more recent studies show that cystine deprivation can rescue cancer cell death resulting from glucose starvation. The intracellular reduction of cystine to cysteine requires NADPH. This causes SLC7A11-high cancer cells to be more dependent on the glucose pentose phosphate pathway (a major provider of NADPH in cells), which in turn is metabolically dependent on glucose (**Figure 2**). Moreover, it has been shown that glucose starvation-induced cell death in SLC7A11-high cancer cells can be prevented by restoring NADPH levels. Altogether, these data suggest that it is NADPH depletion associated with cysteine generation that causes SLC7A11-high cancer cell death under conditions of glucose starvation (**Figure 2**) (57, 58). Various lines of experimental evidence have shown that genetic or pharmacological inhibition of SLC7A11 has a pro-survival role in cancer cells under conditions of glucose starvation. Further, a loss of function screen identified SLC7A11 and SLC3A2 as the top hits for proteins where loss/inactivation provides resistance to glucose starvation (50). Collectively, these studies suggest that enhanced glutamate export and NADPH investment in cancer cells upregulate SLC7A11 levels and enhance dependency on glucose and/or glutamine, though this may be cell line and context dependent (**Figure 2**). Glucose and glutamine abundance are typically limited in the tumor microenvironment (59, 60). Therefore, SLC7A11 overexpression may promote tumor growth/survival but once the tumor is established, SLC7A11-high tumors are more sensitive to glucose or glutamine deficiency in the tumor microenvironment (**Figure 2**).

### Oxidative Stress/Redox Homeostasis

Reactive oxygen species (ROS) are free oxygen radicals such as superoxide $\left({\text{O}}_{2}^{-}\right)$ , hydroxyl radical (OH^-^), and hydrogen peroxide (H_2_O_2_) formed by reduction-oxidation (redox) reactions. ROS and their products can serve as signaling intermediates that contribute to molecular responses involved in normal biological processes, for example, in stem cell renewal, immune responses, and insulin synthesis (61). However, an unbalanced production of ROS can affect normal physiology by damaging DNA, RNA, proteins, and cellular organelles *via* lipid peroxidation and may even result in cell death (4, 61). This occurs due to an insufficiency of detoxifying mechanisms including glutathione stores and antioxidant enzymes (62). This imbalance between ROS production and the antioxidant defense has been implicated in many pathologies including cancer, pulmonary hypertension, retinal damage, and asthma (22, 63–65). SLC7A11 plays an antioxidant role by supporting GSH generation *via* cystine import (4, 9). GSH plays a vital role in many cellular functions including maintaining an intracellular redox balance, reducing hydrogen peroxide or oxygen radicals, detoxification of electrophiles, storing cysteine, and regulating multiple other cellular processes (15, 66). Cancer cells have higher levels of ROS than normal cells, which promotes tumorigenesis, however, too much intracellular ROS triggers cell death and inhibits tumor progression. These increased ROS levels can be induced by irradiation, cytotoxic compounds or inhibition of antioxidants and the antioxidant defense system (67). Oxidative stress in the tumor microenvironment is regulated by SLC7A11 *via* maintenance of the cystine/cysteine redox cycle across the cell membrane. Residual cysteine from GSH synthesis is exported and is rapidly oxidized to cystine. SLC7A11 plays a role in continuous import of this extracellular cystine. This creates a cystine/cysteine redox cycle across the membrane and creates a reducing extracellular environment that supports cancer cell growth and survival (4, 51).

### Cell Death

Ferroptosis is an iron-dependent cell death which is accompanied by iron accumulation and lipid peroxidation. A ferroptosis-associated decrease in antioxidant capacity and accumulation of lipid ROS in cells leads to oxidative cell death. It differs from other forms of cell death in that those cells undergoing ferroptosis have shrunken and dense mitochondria but do not show plasma membrane blebbing, DNA fragmentation, or Caspase-3 cleavage (68). In recent years, ferroptosis has been associated with many pathologies including cancer, neurological diseases, ischemia etc. SLC7A11 overexpression confers resistance to ferroptosis in cancer cells by importing cystine for the synthesis of GSH and by relieving lipid ROS stress by activation of glutathione peroxidase 4 (GPX4) (22). GPX4 catalyzes the reduction of hydrogen and lipid peroxides and protects cells from oxidative stress. Inactivation of GPX4 leads to an accumulation of lipid peroxides and ferroptosis (68–71). Therefore, SLC7A11 prevents cells from undergoing ferroptosis by importing cystine and promoting GSH synthesis. In acute liver failure, overexpression of SLC7A11 plays a protective role in controlling the level of lipopolysaccharide-D-galactosamine-induced hepatocyte acute injury primarily through inhibiting ferroptosis (72). In contrast, SLC7A11 enhances ferroptosis in myofibroblast hepatic stellate cells, which can exacerbate liver injury (73). Escaping cell death is one of the hallmarks of cancer. In 2012, Li et al. showed that a specific mutation in p53 (3KR mutant that cannot be acetylated on certain lysine residues) causes cancer cells to lose the ability to induce cell cycle arrest or apoptosis. However, it could still prevent tumor formation *in vivo* (74). Jiang et al. later discovered that this mutation preserves the tumor suppressive effect of p53 partly by suppressing SLC7A11 expression and inducing ferroptosis (29). Additional p53 mutations also link p53 regulation of SLC7A11 and ferroptosis in cancer (75). Another tumor suppressor, BAP1, frequently found lost or mutated in cancers, has been shown to repress SLC7A11 expression thereby impairing cystine uptake and promoting ferroptosis in cancer cells. Furthermore, SLC7A11 overexpression or treatment with ferroptosis inhibitors has been shown to partly overcome the tumor growth suppression caused by BAP1 restoration in tumors (36, 76, 77). In addition to ferroptosis, SLC7A11 inhibition has been linked to apoptosis in murine melanocytes and cancer cells in a context dependent manner. Unlike erastin (**Figure 1**), SLC7A11 inhibitor HG106 mainly induces apoptosis, likely by GSH depletion (78). The exact mechanism behind the GSH depletion mediated induction of ferroptosis *vs* apoptosis remains to be understood. There may be cell and context dependent differences along with potential off-target effects of the two drugs (34, 79, 80). Therefore, studying the same cell line following treatment with erastin or HG106 may provide insight into the mechanisms underlying these cell death pathways. Studying apoptosis or ferroptosis deficient cells following SLC7A11 inhibition will address some of these unanswered questions.

### Mutant-KRAS and SLC7A11

**Figure 3:**
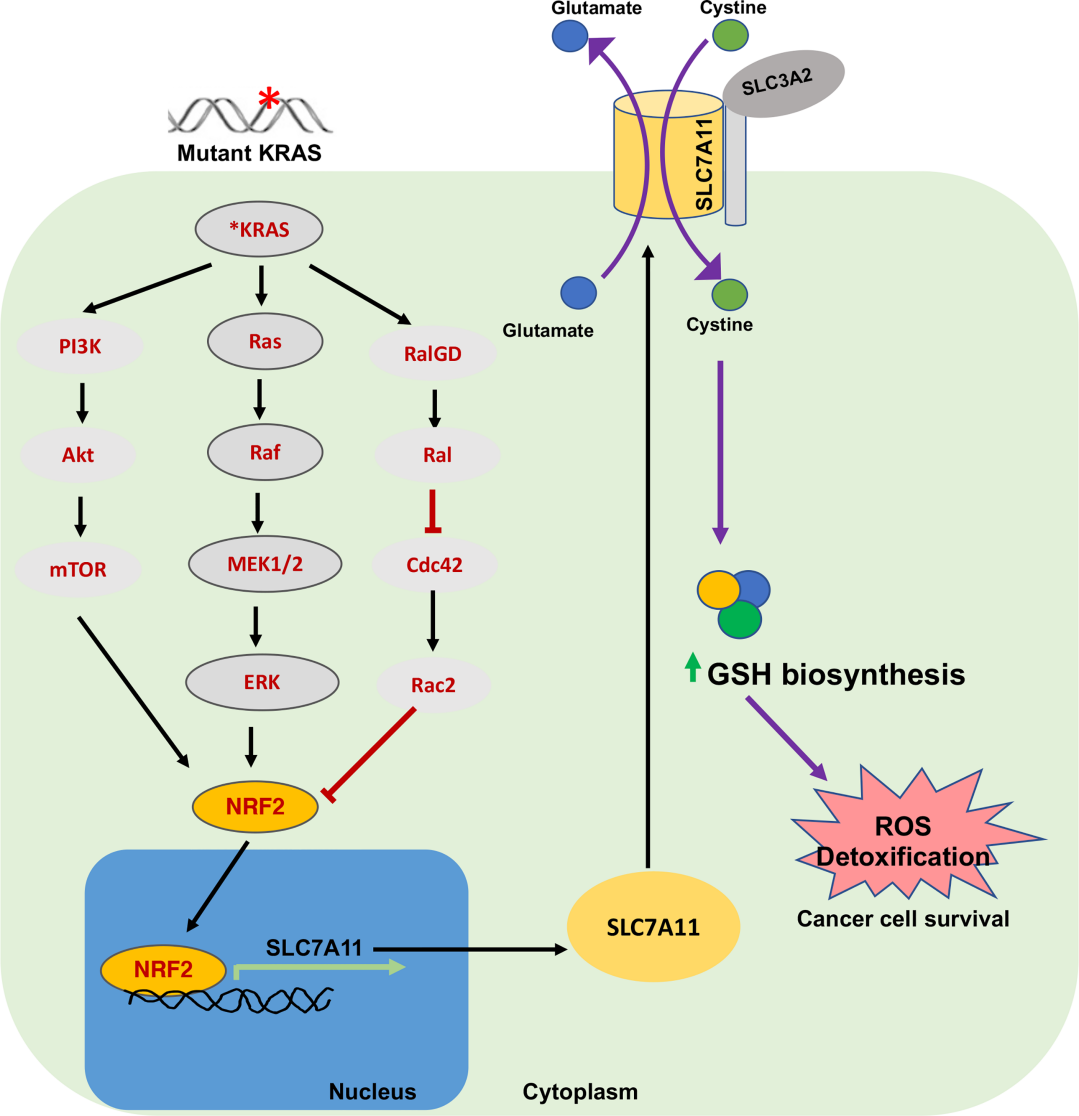
Oncogenic KRAS and SLC7A11 in cancer. Mutations in KRAS induce SLC7A11 overexpression through activation of transcription factor NRF2 *via* downstream signaling pathways including MAPK, PI3K/AKT and RAS-related protein pathways. This regulates expression of SLC7A11 and elevates intracellular cysteine levels and GSH. This signaling plays an important role in sustaining oxidative balance in KRAS-mutant cancer cells and promotes their progression and survival. NRF2, Nuclear erythroid 2-related factor; RAL, RAS-related protein.

*KRAS* is one of the most commonly mutated oncogenes in many cancers (for example, in pancreas and lung). Mutant *KRAS* has recently been shown to promote *SLC7A11* transcription *via* its cooperation with NRF2 and/or ATF4 (**Figure 3**) (56, 78, 81). Furthermore, inhibition of SLC7A11 *via* genetic or pharmacological targeting attenuates mutant-KRAS associated tumor growth in xenograft models *via* induction of lipid peroxidation and ferroptosis (56). It has also been shown in *Slc7a11* deficient mice that oncogenic KRAS-driven pancreatic adenocarcinoma (PDAC) tumor growth is significantly impaired (56). Suppression of xCT was demonstrated to cause synthetic lethality in mutant-KRAS lung adenocarcinoma (78). This suggests that higher ROS levels in mutant-RAS driven cancers enhance dependency on GSH production for survival and that SLC7A11 associated ferroptosis suppression may support mutant-KRAS driven cancer growth. Therefore, targeting or co-targeting the SLC7A11/GSH axis will have therapeutic benefit in mutant-KRAS driven tumors.

### Cancer Immunity and SLC7A11

Several cancer immunotherapies have been shown to inhibit SLC7A11 function and, in turn, induce ferroptosis in tumor cells. For example, programmed death ligand-1 (PD-L1) blockade therapy and CD8+ T cells increase ROS levels by releasing interferon gamma (IFNγ) which promotes the binding of transcription factor STAT1 to the SLC7A11 transcriptional start site, downregulating expression. Similarly, interferon regulatory factor 1 upregulation by IFNγ reduces SLC7A11 transcription through Janus kinase (JAK) (82). Post-immunotherapy radiotherapy synergizes with IFNγ, further promoting ferroptosis *via* SLC7A11 inhibition (83). Suppression of SLC7A11 is also linked to T cell memory-associated enhanced immune responses (22). Therefore, targeting SLC7A11 in combination with immunotherapy may work as an effective treatment strategy for certain cancers.

### Resistance to Cancer Therapy

SLC7A11 overexpression is associated with resistance to immunotherapy, as well as chemo- and radio-resistance, including to drugs such as gemcitabine and cisplatin (84). Enhanced GSH synthesis and inhibition of ferroptosis are believed to promote SLC7A11-associated drug and radio-resistance, respectively. For example, SLC7A11 provides resistance to BRAF and MEK inhibitors in BRAF^V600E^ mutant melanoma, geldanamycin resistance in lung cancer, cisplatin resistance in gastric cancer, temozolomide resistance in glioma and gemcitabine resistance in pancreatic cancer (84–89). Multiple studies have shown that this therapeutic resistance can be reversed by direct targeting of SLC7A11 (90–92). In head and neck cancer, for example, treatment with erastin or sulfasalazine re-sensitizes cancer cells to cisplatin treatment (93).

### Others

While high SLC7A11 expression may be detrimental for patient outcomes in cancer, it also plays a role in healing in many tissues. For example, low expression of SLC7A11 in cardiomyocytes results in reduced levels of GSH, increased ferroptosis, and is associated with cardiomyopathy and cardiac failure in mice (94). Overexpression of STAT3 leads to increased expression of SLC7A11 and plays a protective role in an intestinal ischemia/reperfusion-induced acute lung injury mouse model by reducing ferroptosis (95). It was found that SLC7A11 mediated cystine uptake is crucial for gastric chief cell plasticity and ROS mitigation. CD44v9 stabilizes SLC7A11 for enhanced cystine uptake in chief cells following parietal cell loss during gastric injury (96). SLC7A11 is significantly upregulated during liver regeneration and overexpression in hepatocytes enhances repopulation and recovery following toxic liver injury (97). In contrast to these roles in healing, it was shown by Sprimont and colleagues that ablation of *Slc7a11* in the context of spinal cord injury in mice is beneficial. *Slc7a11* knockout mice have more rapid body weight and motor recovery post-injury (98).

## Roles Identified for SLC7A11 in Non-Gastrointestinal Tract Cancers

SLC7A11 expression correlates with tumor invasion and metastasis in various non-gastrointestinal (non-GI) and gastrointestinal (GI) tract cancers (22). In prostate cancer, SLC7A11 expression is increased in the metastatic stroma and is associated with poor prognosis. *SLC7A11* knockdown leads to an increase in ROS levels, inhibiting tumor cell invasion in the context of co-culture with stromal cells (99). SLC7A11 has also been shown to be important in breast cancer stem cell maintenance and correlates with poor prognosis in patients (55, 100). Overexpression of SLC7A11 provides cancer stem cell-like properties to glioblastoma cells and is related to accelerated tumor growth and enhanced chemoresistance (88). SLC7A11 serves as an independent indicator of poor prognosis in acute myeloid leukemia (101) and an independent risk factor in ovarian cancer (102). SLC7A11 overexpression is linked to enhanced tumor proliferation and progression in melanoma and poor overall survival and advanced pathology in papillary thyroid carcinoma (103, 104). Sulforaphane (a sulfur compound found in many vegetables) induces cell death in small cell lung carcinoma *via* induction of ferroptosis. The levels of SLC7A11 were found to be significantly lower in the sulforaphane treatment group (105). Treatment with anti-SLC7A11 siRNAs induces significant cell death in KRAS-mutant lung adenocarcinoma cells (78). Likewise, SLC7A11 was found to be highly expressed in the cytoplasmic membrane in non-small-cell lung carcinoma (NSCLC) and is associated with poorer prognosis. Treatment of xenografted NSCLC mice with sulfasalazine, a potent inhibitor of SLC7A11, significantly prolonged their survival (21). Administration of SLC7A11 targeting miR-5096 induces ferroptosis in human breast cancer cells and lidocaine treatment results in ferroptosis and ROS induction in both breast and ovarian cancer cells (106). SLC7A11 has been found to be involved in approximately 50% of glioma patient tumors. Intracranially implanted SLC7A11 expressing tumor cells grow faster, produce more glutamate toxicity, induce seizures, and shorten overall survival (107). It was found that SLC7A11- and ferroptosis-related gene signatures (lipid metabolism-associated genes: ACSL4, ALOX5, ACACA, ZEB1, FADS2 and antioxidant metabolism-associated genes: NOX1, GCLM) offer prognostic value in bladder cancer and that targeting these genes is therapeutically beneficial (108).

## Gastrointestinal Tract Cancers

### Esophageal Cancer

Evaluation of 127 esophageal squamous cell carcinoma patients who received radical chemoradiotherapy revealed SLC7A11 and NRF2 overexpression in the tumor tissue. Overexpression of SLC7A11 was associated with lymph node metastasis and shorter overall survival in these patients (109).

### Liver Cancer

Higher levels of SLC7A11 are associated with poor differentiation and advanced disease stage in hepatocellular carcinoma (HCC) (110, 111). Analysis of clinical data from The Cancer Genome Atlas (TCGA) showed that SLC7A11 overexpression correlates with poor outcome in liver cancer (112). SLC7A11 knockdown prevents HCC growth (113). Interleukin-1β induces SLC7A11 expression in HCC cells and is associated with HIF1α levels. This αKG- HIF1α cascade upregulates PD-L1 and colony-stimulating factor-1 (CSF1), which further increase HCC metastasis *via* infiltration of tumor associated macrophages (TAMs) and myeloid derived suppressor cells (MDSCs). Either depletion of TAMs or MDSCs, treatment with a CSF1 inhibitor, an anti-PD-L1 antibody or Anakinra (an IL1β inhibitor), or SLC7A11 knockdown, all result in a reduction in HCC metastasis (114). xCT inhibition by sulfasalazine or through siRNA targeting SLC7A11 leads to reduced cell proliferation *in vitro* and *in-vivo via* reduction in GSH levels and an increase in ROS by inhibiting the CD44v9-SLC7A11 interaction (111, 113).

### Gastric Cancer

Reduction of SLC7A11 expression using pharmacological inhibitors (erastin, Jiyuan Oridonin A derivative a2, levobupivacaine), miRNA (miR-375), long non-coding RNA and siRNA has been shown to inhibit tumor progression through induction of ferroptosis (115–118). Treatment with miR-375 was shown to reduce the stemness of gastric cancer stem cells (116). A local anesthetic, levobupivacaine, induces ferroptosis in gastric cancer and suppresses cell proliferation both *in vitro* and *in vivo* by elevating the levels of miR-489-3p and suppressing SLC7A11 (117). Decreased expression of long non-coding RNA SLC7A11-AS1 contributes to tumor progression and is a poor prognostic indicator in gastric cancer patients (118). Tanshione IIA, an active compound isolated from the rhizome of Chinese herb Danshen has been shown to block gastric cancer stem cells *via* ferroptosis induction and SLC7A11 downregulation (119). SLC7A11 inhibition *via* sulfasalazine reduced colony formation, proliferation, metastasis, and invasion of gastric cancer cells *in vitro* (120).

### Colorectal Cancer

Analysis of 21 different types of cancer in 7462 cancer samples showed that both SLC7A11 and GPX4 are overexpressed in colorectal cancer (CRC) (121). Elesclomol, a copper chelator, suppresses CRC both *in vitro* and *in vivo* by inducing ROS accumulation and is associated with downregulation of SLC7A11 protein levels *via* ubiquitination and degradation (122). CRC stem cells have higher levels of cysteine, GSH, and SLC7A11 and, therefore, exhibit lower levels of ROS as compared to CRCs. Genetic and pharmacological inhibition of SLC7A11 in CRC stem cells significantly attenuates their viability *in vitro* and *in vivo* in comparison to CRCs (123, 124). Using two different cell lines and a xenograft mouse model of CRC, it was shown that 2-Imino-6-methoxy-2H-chromene-3-carbothioamide (IMCA), a benzopyran derivative, downregulates SLC7A11 levels leading to increased ROS and ferroptosis in CRC cells. IMCA treatment reduces CRC cell viability *in vitro*, inhibits the growth of xenografted CRC cells *in vivo*, and is associated with a decrease in the phosphorylation of mTOR and its downstream target P70S6K (125).

### Pancreatic Cancer

Pancreatic ductal adenocarcinoma (PDAC) is characterized by a poor 5-year survival rate (~10%) due to inherent chemoresistance, barriers to drug delivery, and a lack of early diagnostics. SLC7A11 expression was found to be upregulated in many pancreatic cancer cell lines (112). Recently, Badgley et al., demonstrated a role for SLC7A11 in PDAC. More than 90% of PDAC cases harbor mutations in KRAS and mutant KRAS signaling is linked to increased ROS production and, therefore, increased dependency on cystine import *via* SLC7A11. Culturing PDAC cell lines in cystine deficient conditions induces cell death in more than 80% of cells, which is rescued by the addition of antioxidant Trolox. Addition of system ${\text{x}}_{\text{c}}^{-}$ inhibitor imidazole ketone erastin (IKE) has similar effects on the cell viability. Cysteine starved PDAC cells undergo plasma membrane destabilization and show significantly increased levels of lipid peroxidation, a signature of cells undergoing ferroptosis. Pharmacological or genetic inhibition of SLC7A11 induces ferroptosis and blocks PDAC growth in a genetically engineered mouse model of PDAC (56). Daher et al. showed that xCT knockout in two different PDAC cell lines (MIA PaCa-2 and CAPAN-2) induces ferroptosis and significantly affects growth and proliferation *in vitro* and *in vivo* when injected subcutaneously in nude mice (126). Gene ontology analysis of 43 different ferroptosis regulators revealed that both SLC7A11 and SLC3A2 are upregulated in pancreatic cancer samples and are associated with gemcitabine resistance (64). Bioinformatic analysis of PDAC samples from The Cancer Genome Atlas (TCGA) found that lower expression of miR-139-5p, along with higher expression of SLC7A11 is associated with poor clinical outcomes. Overexpression of miR-139-5p is associated with suppression of SLC7A11 in PDAC cells and can be exploited for inhibition of cell proliferation, invasion, and metastasis (127). One of the major contributors to PDAC progression and metastasis are cancer-associated fibroblasts (CAFs), which block drug delivery and create a hypoxic microenvironment. Sharbeen et. al., found that PDAC-associated CAFs are heavily dependent on SLC7A11 for cystine uptake and GSH synthesis to balance ROS and oxidative stress in tumors and, therefore, support PDAC tumor progression. High stromal SLC7A11 levels in human PDAC samples are predictive of poor overall survival independent of SLC7A11 expression in the tumor cells themselves. SLC7A11 knockdown in CAFs inhibited proliferation in both SLC7A11^low^ and SLC7A11^high^ PDAC and increased sensitivity to oxidative stress and ferroptosis. Furthermore, treatment with nanoparticle-siRNA targeting SLC7A11 significantly decreased PDAC tumor growth (23).

## Targeting SLC7A11 in Cancer

Thus far, SLC7A11 targeting strategies include either directly inhibiting SLC7A11 transporter activity or indirectly targeting SLC7A11-associated metabolic susceptibilities and pathways in cancer. Direct targeting of SLC7A11 includes the use of inhibitors: sulfasalazine (128, 129), erastin (130, 131), imidazole ketone erastin (IKE) (132), sorafenib (133, 134), and HG106 (78). These inhibitors induce ferroptosis by blocking cystine uptake *via* SLC7A11 and fall under the category of class I ferroptosis inducers. However, each one has its pros and cons. For example, due to unfavorable pharmacological properties, sulfasalazine treatment does not result in better outcomes in PhaseI/II clinical trials. However, when delivered as a zinc oxide-sulfasalazine nanoparticle derivative, it has better tumor retention with improved cytotoxic effects and no evident damage to healthy cells (135). As a system ${\text{x}}_{\text{c}}^{-}$ inhibitor and ferroptosis inducer, the therapeutic potential of erastin has been shown in multiple cancer types including breast cancer (130). However, erastin is poorly soluble and metabolically unstable *in vivo* and, therefore, cannot be used for clinical studies. An analog, IKE, is metabolically stable and was shown to be effective in genetically engineered mouse models of PDAC, however, it has not yet been tested in the clinic. Both sorafenib and sulfasalazine are FDA approved drugs that have been shown to induce ferroptosis and inhibit tumor growth, however, in addition to inhibiting SLC7A11, sorafenib acts as a multi-kinase inhibitor and sulfasalazine blocks prostaglandin production (136, 137). Because of these off target effects, both are associated with adverse clinical events (128, 138). Therefore, targeting metabolic vulnerabilities associated with SLC7A11 may prove to be a better therapeutic strategy against cancer.

As previously mentioned, SLC7A11^high^ cancer cells are more vulnerable to glucose and glutamine starvation. CB-839, a glutaminase inhibitor, has been shown to potently suppress tumor progression in KEAP1-mutant *vs.* wild type patient-derived xenografts. KEAP1 mutation/inactivation leads to NRF2 stabilization and results in the upregulation of NRF2-target genes including SLC7A11. KEAP1-mutant lung cancers are characterized by high SLC7A11 expression levels (139, 140). Similarly, KL-11743, a GLUT1/3 dual inhibitor selectively inhibits SLC7A11^high^ tumor growth in cell lines and in patient derived xenografts (141). High-throughput screening of a number of compounds that inhibit glutamate export in triple negative breast cancer cells suggests that capsazepine (CPZ) can effectively inhibit SLC7A11 and increase intracellular ROS (142). Similarly, in a screen of mutant KRAS lung adenocarcinoma cells, HG106 was found to specifically inhibit SLC7A11 function and decrease tumor burden *in vivo via* ROS induction and mitochondrial and endoplasmic reticulum dysfunction (78). Various glutamate analogues (L-Homocysteate, L-Quisqualate, 4-bromo-homoibotenate and S-4-Carboxy-phenylglycine (CPG)) have been shown to affect the exchange between L-cystine and glutamate across the cell membrane through system ${\text{x}}_{\text{c}}^{-}$ (143). SLC7A11 activity can also be affected indirectly by targeting its upstream regulators. For example, MEK inhibitor AZD6244 and BAY-11-7085 inhibit NRF2, JQ-1 targets BD4, AG879 targets receptor tyrosine kinase TrkA, and all indirectly inhibit SLC7A11 expression (22). Paclitaxel enhances ferroptosis by inhibiting SLC7A11 transcription (144). Various immunotherapy approaches that reduce SLC7A11 expression *in vivo* have promise. For example, anti-SLC7A11 DNA vaccines that utilize plasmids to express full length SLC7A11 have been shown to induce regression in lung metastases in 4T1-tumor bearing mice (100). Virus-like particles (AX09-0M6) displaying the 6^th^ extracellular loop of SLC7A11 common to both mouse and human SLC7A11 disabled the self-renewal ability of breast cancer stem cells (145). Finally, a bovine herpesvirus 4-based vector delivering full length SLC7A11 DNA protected mice from breast cancer metastases by targeting cancer stem cells (146).

## Future Perspectives

The redox status of cancer cells reflects many aspects of carcinogenesis including cell growth, proliferation, migration, metabolism, invasion, and metastases. Cancer progression and tissue injury are often associated with a state of redox imbalance. To survive under conditions of increased oxidative stress, cancer cells adopt various strategies to produce antioxidants, for example, upregulation of SLC7A11. SLC7A11 functions in a variety of roles in cancer including metabolic reprogramming, nutrient dependency, growth, proliferation, invasion, and drug resistance (**Figure 1**). As SLC7A11 is dispensable in normal, healthy cells and *Slc7a11* knockout mice are viable with no associated pathologies, targeting SLC7A11 poses a promising therapeutic target. In recent years, the role of SLC7A11 has been extensively studied in a variety of cancers including both GI and non-GI tract cancers. However, there are many questions that remain to be answered. A better understanding of the cell and context dependent role of SLC7A11 under different conditions of nutrient availability (for example, glucose and glutamine limiting conditions) is needed (**Figure 2**). There are multiple studies that highlight the various transcriptional and epigenetic factors that regulate SLC7A11 expression, however, further studies are required to understand whether SLC7A11 is also regulated *via* other mechanisms, especially post translational mechanisms and the existence of key interaction partners, etc. It would be interesting to elucidate the contribution of SLC7A11 to ferroptosis *versus* other forms of cell death (apoptosis, necrosis, autophagy) following inhibition in a variety of cancers. Identifying novel targets (synthetically lethal partners, metabolic vulnerabilities, immunotherapies, chemotherapies, signaling pathway inhibitors, upstream or downstream targets) that can be co-targeted with SLC7A11 inhibition in cancer may significantly improve the anti-cancer effects. For example, because of higher dependency of SLC7A11-high cancers on glucose and glutamine, combination targeting strategies including drugs/inhibitors that target/block glucose or glutamine metabolism would increase therapeutic efficacy. *Slc7a11* knockout and transgenic mouse models are ideal to study the role of SLC7A11 in tumor progression and maintenance. All currently available SLC7A11 inhibitors, including sulfasalazine and erastin, have either off-target effects or bioavailability issues. Utilizing biocompatible nanoparticles to package and deliver these drugs would enhance their efficacy, increase their bioavailability, and potentially eliminate associated side effects. Therefore, more selective SLC7A11 inhibitors and genetic targeting strategies, including small interfering RNAs (siRNA) and small guide RNAs (sgRNA), would be ideal for clinical purposes.

## Author Contributions

NJ conducted the research and wrote/edited the manuscript. KT researched the manuscript. KD edited the manuscript, supervised, and funded the research. All authors contributed to the article and approved the submitted version.

## Funding

The DelGiorno laboratory is supported by the Vanderbilt-Ingram Cancer Center Support Grant (NIH/NCI 5P30 CA068485), the Vanderbilt-Ingram Cancer Center SPORE in Gastrointestinal Cancer (NIH/NCI 5P50 CA236733), a Vanderbilt Digestive Disease Research Center Pilot and Feasibility Grant (NIH/NIDDK 5P30 DK058404), an American Gastroenterological Association Research Scholar Award (AGA2021-13-02), NIH/NIGMS 1R35 GM142709, and the Sky Foundation, Inc. (AWD00000079).

## Conflict of Interest

The authors declare that the research was conducted in the absence of any commercial or financial relationships that could be construed as a potential conflict of interest.

## Publisher’s Note

All claims expressed in this article are solely those of the authors and do not necessarily represent those of their affiliated organizations, or those of the publisher, the editors and the reviewers. Any product that may be evaluated in this article, or claim that may be made by its manufacturer, is not guaranteed or endorsed by the publisher.
